# *Staphylococcus aureus* Produces Membrane-Derived Vesicles That Induce Host Cell Death

**DOI:** 10.1371/journal.pone.0027958

**Published:** 2011-11-16

**Authors:** Mamata Gurung, Dong Chan Moon, Chi Won Choi, Jung Hwa Lee, Yong Chul Bae, Jungmin Kim, Yoo Chul Lee, Sung Yong Seol, Dong Taek Cho, Seung Il Kim, Je Chul Lee

**Affiliations:** 1 Department of Microbiology, Kyungpook National University School of Medicine, Daegu, Korea; 2 Division of Life Science, Korea Basic Science Institute, Daejeon, Korea; 3 Department of Oral Anatomy and Neurobiology, School of Dentistry, Kyungpook National University, Daegu, Korea; Charité-University Medicine Berlin, Germany

## Abstract

Gram-negative bacteria produce outer membrane vesicles that play a role in the delivery of virulence factors to host cells. However, little is known about the membrane-derived vesicles (MVs) produced by Gram-positive bacteria. The present study examined the production of MVs from *Staphylococcus aureus* and investigated the delivery of MVs to host cells and subsequent cytotoxicity. Four *S. aureus* strains tested, two type strains and two clinical isolates, produced spherical nanovesicles during *in vitro* culture. MVs were also produced during *in vivo* infection of a clinical *S. aureus* isolate in a mouse pneumonia model. Proteomic analysis showed that 143 different proteins were identified in the *S. aureus*-derived MVs. *S. aureus* MVs were interacted with the plasma membrane of host cells via a cholesterol-rich membrane microdomain and then delivered their component protein A to host cells within 30 min. Intact *S. aureus* MVs induced apoptosis of HEp-2 cells in a dose-dependent manner, whereas lysed MVs neither delivered their component into the cytosol of host cells nor induced cytotoxicity. In conclusion, this study is the first report that *S. aureus* MVs are an important vehicle for delivery of bacterial effector molecules to host cells.

## Introduction

*Staphylococcus aureus* causes a wide range of diseases in humans, from uncomplicated local infection to life threatening systemic infection, both in health care facilities and the community [1]. Most staphylococcal infections can be successfully treated with antibiotics, but strains resistant to vancomycin have emerged, which is of great concern in clinical setting [2]–[4]. The ability of *S. aureus* to cause a wide range of diseases is associated with various structural components such as the capsule, peptidoglycan, teichoic acid, and protein A, toxins such as cytotoxins, exfoliative toxins, enterotoxins, and toxic shock syndrome toxin-1 (TSST-1), and enzymes such as coagulase, catalase, hyaluronidase, fibrinolysin, lipase, and nuclease [1], [5]–[7]. However, the secretion of these virulence determinants from *S. aureus* and their delivery to host cells has not been fully characterized. Lee *et al*. [8] recently demonstrated that *S. aureus* produced membrane-derived vesicles (MVs) during *in vitro* culture and many virulence-associated proteins were identified in the *S. aureus* MVs. Therefore, it is possible that *S. aureus* MVs play a role in the delivery of virulence factors to host cells, which is similar to that of the outer membrane vesicles (OMVs) of Gram-negative bacteria [9].

A variety of Gram-negative pathogenic or environmental bacteria secretes OMVs, which are produced during normal bacterial growth [10]. OMVs are spherical and bilayered nanovesicles with an average diameter of 20–300 nm. They are composed of lipopolysaccharides, phospholipids, outer membrane proteins, periplasmic proteins, cytosolic proteins, and even nucleic acids [9], [11]–[13]. In addition, virulence factors, like cytolysin A in *Escherichia coli* [14], heat-labile toxin in enterotoxigenic *E. coli* [15], hemolytic phospholipase C and alkaline phosphatase in *Pseudomonas aeruginosa* [16], and leukotoxin in *Actinobacillus actinomycetemcomitans* [17], have been identified, and their contributions to bacterial pathogenesis have been characterized. In contrast, little is known about such processes in Gram-positive bacteria. MV production has been reported in *S. aureus*, *Bacillus anthracis*, *B. cereus*, and *B. subtilis* [8], [18]–[19], and more recently *S. aureus* MVs have been shown to induce atopic dermatitis-like inflammation [20]. However, the production of MVs from Gram-positive bacteria during *in vivo* infection and their direct associations with host cell pathology have not been determined.

The present study examined the production of MVs from a clinical *S. aureus* isolate during *in vivo* infection and further investigated the delivery of bacterial effector molecules to host cells via MVs and subsequent cytotoxicity. We report that *S. aureus* produces MVs during *in vivo* infection and also that *S. aureus* MVs play a role in the delivery of bacterial effector molecules to host cells.

## Results

### Production of MVs from *S. aureus*

**Figure 1:**
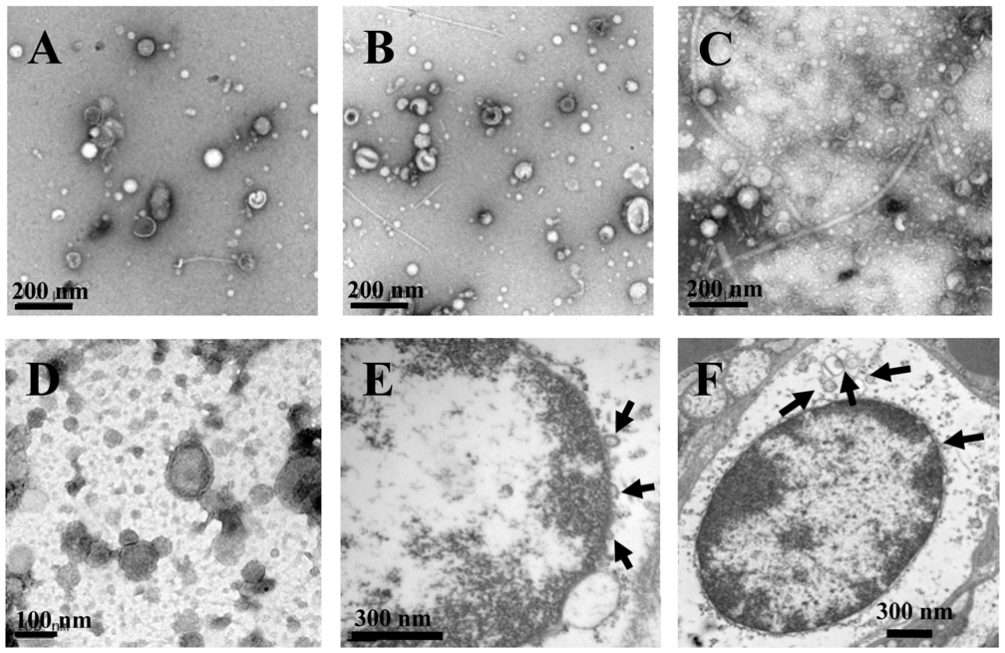
Production of MVs from *S. aureus*. (A to D) Transmission electron micrograph of MVs prepared from S. aureus ATCC 25923 (A), ATCC 700699 (B), 103D (C), and 06ST1048 (D) cultured in LB broth. (E and F) Production and secretion of MVs from S. aureus 06ST1048 during in vivo infection. Neutropenic mice were infected with 1×10^7^ CFU of bacteria intratracheally. The S. aureus-infected mice were sacrificed 18 h after bacterial injection. Arrows indicate the spherical nanovesicles from S. aureus.

Lee *et al*. [8] were the first to demonstrate that *S. aureus* ATCC 14458 produced MVs during *in vitro* culture. However, MV production was not reported in other *S. aureus* strains. To determine whether MV production is common feature of *S. aureus*, two type strains, ATCC 25923 (control strain for antimicrobial susceptibility test) and ATCC 700699 (vancomycin-intermediate *S. aureus* Mu50), and two clinical isolates, TSST-1 producing *S. aureus* 103D and methicillin-resistant *S. aureus* (MRSA) 06ST1048, were cultured in Luria-Bertani (LB) broth and the extracellular vesicles were collected from the culture supernatants. Transmission electron microscopy (TEM) analysis showed that all *S. aureus* strains tested produced MVs during *in vitro* culture. *S. aureus*-derived vesicles were spherical and bilayered structures with the diameters of approximately 20 to 130 nm (Fig. 1A-1D). Next, in order to determine whether *S. aureus* produced MVs during *in vivo* infection, mice were infected intratracheally with *S. aureus* 06ST1048 and sacrificed 18 h after bacterial administration. Histological examination showed edema, hemorrhage, and infiltration of polymorphonuclear cells in the tissues of both lungs (data not shown), suggesting the occurrence of pneumonia. TEM analysis of the infected lung tissues revealed the budding of spherical nanovesicles from bacterial surfaces (Fig. 1E). Moreover, the secreted MVs were observed in the surrounding milieu (Fig. 1F). These results indicate that *S. aureus* produces and secretes MVs into extracellular milieu during both *in vitro* culture and *in vivo* infection.

### Identification of proteins in *S. aureus* MVs

**Figure 2:**
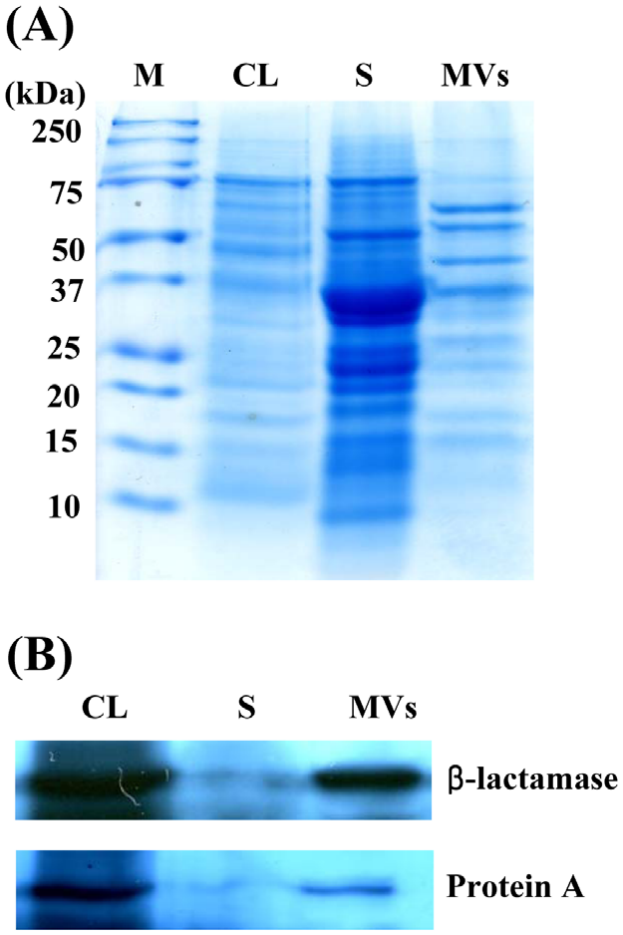
Proteins identified in the S. aureus-derived MVs. SDS-PAGE of proteins packaged in the MVs from S. aureus 06ST1048 (A) and its Western blot analysis (B). The samples were separated on 12% SDS-PAGE and immunoblotted with anti-protein A and anti-β-lactamases antibodies. Lanes M, molecular weight maker; CL, bacterial cell lysate fraction; S, supernatant fraction; and MVs, membrane-derived vesicle fraction.

To analyze proteins packaged in the *S. aureus* MVs, the purified MVs from *S. aureus* 06ST1048 were subjected to sodium dodecyl sulfate-polyacrylamide gel electrophoresis (SDS-PAGE). Many protein bands were identified in the MV fraction, but the positions of major bands were different in the culture supernatant and the MV fraction (Fig. 2A). To identify proteins packaged in the *S. aureus*-derived MVs, proteomic analysis was performed. A total of 143 proteins were identified in the MVs derived from *S. aureus* 06ST1048 (Table S1). A putative pyruvate dehydrogenase E1 component, beta subunit (D1GS80) with the expected molecular mass of 35.2 kDa was detected in the highest abundance, followed by dihydrolipoamide acetyltransferase component of pyruvate dehydrogenase complex (D1GS81) with the expected molecular mass of 46.4 kDa. The identified proteins were classified into five groups based on the location of proteins in the bacteria: cytoplasm (n = 96), cytoplasmic membrane (n = 28), cell wall (n = 3), extracellular region (n = 12), and unknown localization (n = 4). Based on the proteome results from *S. aureus*-derived MVs, Western blot analysis was performed to detect β-lactamases in the MVs. β-lactamases were identified in MVs from *S. aureus* 06ST1048 isolate (Fig 2B). To determine whether the β-lactamases packaged in the MVs were biologically active enzymes, the purified MVs were incubated with the chromogenic cephalosporin nitrocefin, and hydrolysis of the β-lactamase substrate was monitored [21]. This revealed β-lactamases in the MVs degraded nitrocefin and resulted in a brown colorization of nitrocefin, thereby suggesting that biologically active β-lactamases were secreted from bacteria via the MVs. To identify cell wall-associated proteins in the MVs, Western blot analysis of immunoglobulin G-binding protein (protein A), a specific membrane protein of *S. aureus*, was performed. *S. aureus* MVs contained protein A with a molecular size of 42 kDa (Fig 2B). These results suggest that many bacterial proteins, including biologically active proteins, are packaged in the *S. aureus* MVs.

### Delivery of MV components to host cells

**Figure 3:**
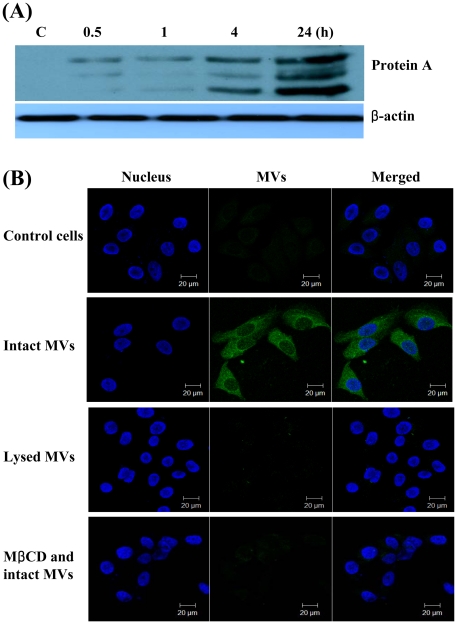
Delivery of protein A packaged in the S. aureus MVs to host cells. (A) Delivery of protein A to host cells via the MVs. HEp-2 cells were treated with S. aureus MVs (20 µg/ml of protein concentrations) for the indicated times. Cell lysates were separated on 12% SDS-PAGE, transferred to membranes, and immunoblotted with anti-protein A and β-actin antibodies. Both full-length and degraded forms of protein A were appeared. (B) HEp-2 cells were treated with intact or lysed MVs (20 µg/ml of protein concentrations) for 24 h. HEp-2 cells were pretreated with 10 mM MβCD for 45 min at 37°C. The cells were fixed, permeabilized, and stained with a rabbit anti-protein A antibody, followed by Alexa Fluor® 488-conjugated rabbit IgG (green). DAPI was used to stain the nuclei (blue). The analytical sectioning was performed from the top to the bottom of the cells. The figure represents all projection of sections in one picture.

To determine the delivery of bacterial components to host cells via the MVs, the time obligation for the entry of protein A, a documented MV protein, in HEp-2 cells was examined by Western blot. Protein A was first detected in the cells within 30 min, and then intact and degraded forms were observed at 24 h after treatment of the MVs (Fig. 3A). To determine whether MVs played a role in the delivery of bacterial effector molecules to host cells, HEp-2 cells were treated with intact MVs and MVs lysed with ethylenediaminetetraacetic acid (EDTA) for 24 h. Cells were permeabilized with Triton X-100 and treated with anti-protein A antibody, followed by Alexa Fluor 488 for protein A. Green fluorescence was noted principally in the cytosolic compartment of the cells treated with intact MVs, whereas the fluorescent intensity of protein A was not observed in the cells treated with lysed MVs (Fig. 3B). These suggest that MVs are a vehicle for the delivery of bacterial components to host cells. In the next series of experiments, we determined the delivery mechanism of MV components to host cells. Since OMVs from Gram-negative bacteria were interacted with the plasma membrane of host cells via lipid rafts [10], [22], HEp-2 cells were pretreated with a cholesterol-destroying agent, methyl-β-cyclodextrin (MβCD), and then treated with *S. aureus* MVs for 24 h. This revealed that MβCD abolished the entry of the MV component, protein A, in host cells (Fig. 3B), thereby indicating that MV components enter the cytosol of host cells via a cholesterol-rich membrane microdomain.

### Host cell death induced by *S. aureus* MVs

**Figure 4:**
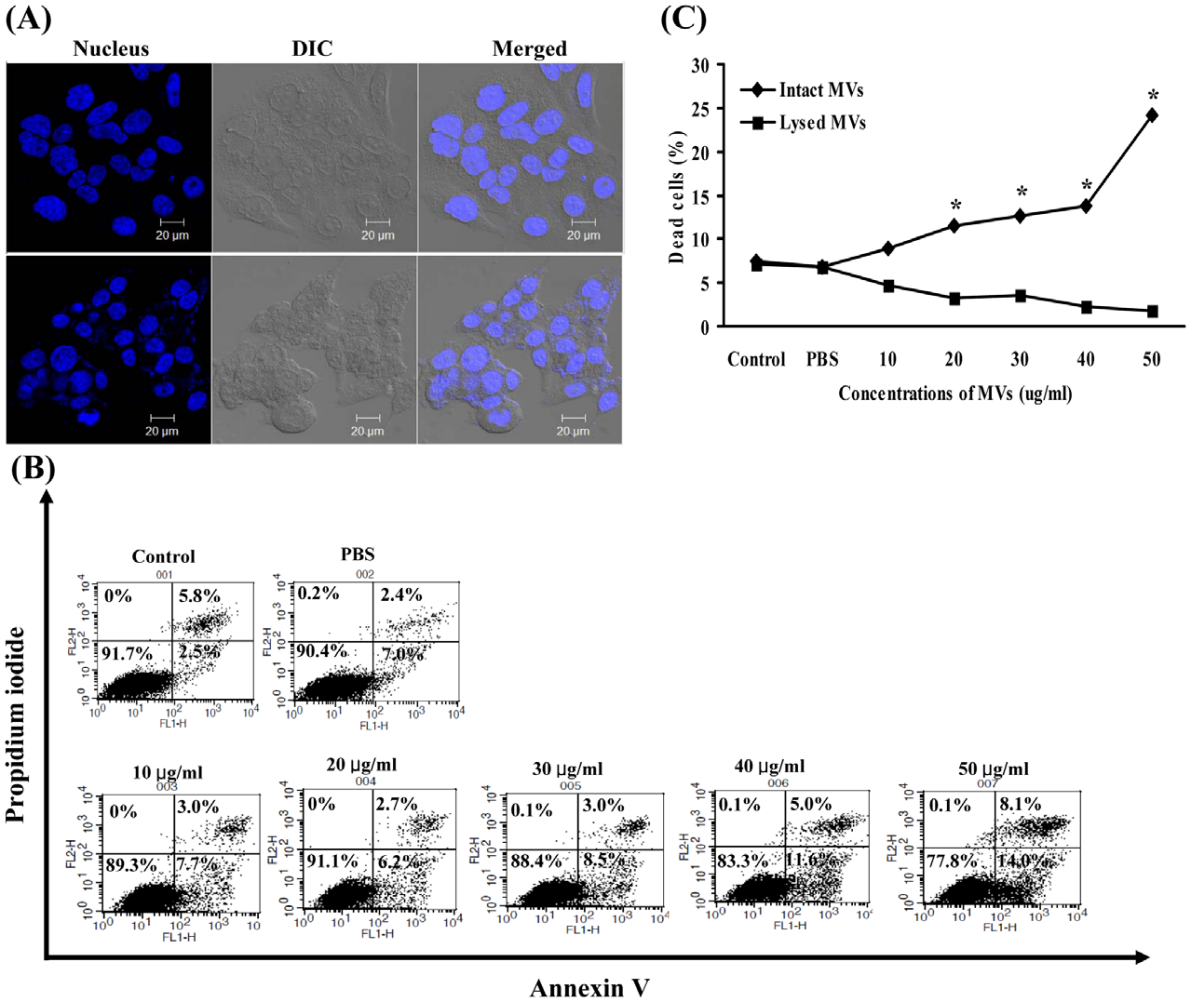
Host cell death induced by the *S. aureus* MVs. (A) HEp-2 cells were treated with 50 µg/ml of MVs for 24 h and stained with DAPI. Upper and lower panels are untreated control cells and S. aureus MV-treated cells, respectively. S. aureus MVs induced host cell pathology, such as condensation of nuclei, nuclear fragmentation and cellular shrinkage. DIC, differential interference contrast microscopy. (B) Flow cytometric analysis of cell death induced by the S. aureus MVs. Upper panel, control cells without MVs and with PBS for 24 h. Lower panel, HEp-2 cells were treated with various concentrations of S. aureus MVs for 24 h. Representative data from three independent experiments are shown. In the graph, cells in right upper and lower parts represent apoptotic cells, and cells in left upper part represent necrotic cells. (C) HEp-2 cells were treated with various concentrations of intact MVs or MVs lysed with EDTA for 24 h. Cells were stained with Annexin V and PI, and then 10^4^ cells were analyzed by flow cytometry. All dead cell population, including Annexin V^+^/PI^+^, Annexin V^+^/PI^−^ and Annexin V^−^/PI^+^ fractions, were calculated. Data are presented as mean ± standard error in three independent experiments. *Statistically significant at p<0.05 using a Student's t-test.

In order to determine whether MVs from *S. aureus* 06ST1048 induced host cell pathology, HEp-2 cells were treated with various concentrations of MVs for 24 h. The morphology of HEp-2 cells treated with 50 µg/ml of MVs showed cellular shrinkage, nuclear condensation, and nuclear fragmentation (Fig. 4A), suggesting that *S. aureus* MVs induce host cell death. To analyze the cell death mechanism induced by the *S. aureus* MVs, HEp-2 cells were treated with various concentrations of MVs for 24 h and then stained with Annexin V and propidium iodide (PI). Flow cytometric analysis showed that *S. aureus* MVs induced apoptotic cell death (Annexin V^+^/PI^−^ and Annexin V^+^/PI^+^ fractions) (Fig. 4B). The cytotoxic effect on HEp-2 cells was statistically significant at a concentration of ≥20 µg/ml of MVs (p<0.05) and was apparently dose-dependent (Fig. 4C). To determine whether lysed MVs induced cytotoxicity, HEp-2 cells were treated with various concentrations of lysed MVs for 24 h. Unlike intact MVs, lysed MVs did not induce cytotoxicity (Fig. 4C). Pretreating HEp-2 cells with MβCD and then treating them with 50 µg/ml of MVs for 24 h did not induce cytotoxicity (9.2±2.6%), although the same concentration of MVs did induce cytotoxicity (24.1±1.7%). These results suggest that the delivery of MV components to cytosol of host cells is prerequisite for cytotoxicity.

## Discussion

Membrane-derived vesicle formation and the delivery of virulence determinants to host cells via these vesicular structures appear to be a conserved process among pathogenic Gram-negative bacteria and eukaryotic pathogens [9], [10], [23]. However, the production of MVs from Gram-positive bacteria during infection and their contribution to bacterial pathogenesis have not been determined. In the current study, we demonstrate for the first time that Gram-positive bacterium, *S. aureus*, produces and secretes MVs into extracellular milieu during *in vivo* infection. *S. aureus* MVs serve as a transport system for virulence-associated components, as demonstrated by host cell death induced by intact MVs, but not by lysed MVs. Thus, our results extend the role of extracellular vesicles in bacterial pathogenesis to Gram-positive bacteria.

The production of MVs was previously identified in *S. aureus* ATCC 14458 [8], but not in other *S. aureus* strains. In the present study, we demonstrate that MV production is a common feature of *S. aureus*. Despite the structural differences between Gram-positive and Gram-negative bacteria, the morphological characteristics of MVs from Gram-positive bacteria shared many common features with OMVs of Gram-negative bacteria [8]. OMVs were spherical and bilayered structures with an average diameter of 20–300 nm [24]–[27]. Similarly, MVs shed from *S. aureus* and *B. anthracis* were bilayered spherical vesicles [8], [18], but the size of MVs from Gram-positive bacteria was relatively smaller than that of OMVs from Gram-negative bacteria. Several studies have demonstrated that a membranous structure of extracellular vesicles from both Gram-positive and Gram-negative bacteria provides protection for effector molecules like toxins, enzymes, and DNA from the extracellular harsh environment and delivers them to target molecules or cells efficiently [18], [28]–[32]. In this study, the biologically active β-lactamases were detected in the MVs of a clinical *S. aureus* isolate as well as MVs of *S. aureus* ATCC 14458 [8], thus allowing the concentrated delivery of β-lactamases to target antimicrobial agents via the MVs. In addition, intact MVs efficiently delivered the MV component, protein A, to host cells, whereas the protein A from lysed MVs did not enter the cytosol of host cells. These results suggest that *S. aureus* MVs are an important vehicle for the delivery of bacterial effector molecules to host cells, a function which may contribute to pathogenesis of *S. aureus* during infection.

The contribution of OMVs to bacterial pathogenesis has been confirmed in a variety of pathogenic Gram-negative bacteria [33]–[37], but the role of MVs in the pathogenesis of Gram-positive bacteria was not fully determined. Rivera *et al*. [18] recently reported that *B. anthracis* secreted toxin components, protective antigen, lethal factor, and edema factor, and anthrolysin via the MVs [18]. Lee *et al*. [8] also demonstrated that *S. aureus* MVs contained extracellular and membrane-associated virulence factors, including toxins, adhesins, proteolysin, coagulase, and other tissue destruction enzymes. Virulence-associated proteins, such as toxins lethal to host cells, extracellular proteases, bacterial adherence and invasion factors in host cells, and immune evasion proteins, accounted for 11% of the 90 protein components identified in the MVs from *S. aureus* ATCC 14458, whereas proteins associated with information storage and processing and with metabolism accounted for 55% [8]. Moreover, another report from the same group showed that *S. aureus* MVs produced proinflammatory mediators, such as interleukin-6, thymic stromal lymphopoietin, macrophage inflammatory protein-1α, and eotaxin, and induced atopic dermatitis-like skin inflammation [20]. In the present study, we identified 143 different proteins in the *S. aureus*-derived MVs. The cytoplasmic proteins were the most abundant (67.1%), followed by membrane proteins (19.6%). Pathogenesis-associated proteins, including IgG-binding protein, ferritin, putative ferrichrome-binding lipoprotein precursor, ABC transporter extracellular binding protein, and lipoprotein for high-affinity iron ion transport, and proteins associated with antibiotic resistance, including β-lactamase, penicillin-binding protein 2, and membrane protein oxaA, were found in the *S. aureus* MVs. These results suggest that bacterial effectors packaged in the MVs are directly associated with bacterial pathogenesis.

Nothing has been reported regarding the interaction of *S. aureus* MVs with host cells and subsequent cellular pathology. The OMVs from Gram-negative bacteria could interact with host cells via either a receptor-mediated endocytic pathway or fusion with the host cell plasma membrane [10], [38]. Bomberger *et al*. [16] showed that OMVs secreted by *Pseudomonas aeruginosa* delivered multiple virulence factors directly into the cytoplasm of host cells by fusion of OMV with lipid rafts in the plasma membrane. They therefore proposed that secreted virulence factors were not released individually, but that bacterial derived OMVs delivered these factors simultaneously [16]. Our previous study on *Acinetobacter baumannii* OMVs also showed that a cholesterol-rich membrane microdomain was required for the delivery of outer membrane protein A packaged in *A. baumannii* OMVs to the cytosol of host cells [22]. Interestingly, the present study found a similar mechanism, in which *S. aureus* MVs delivered MV components to host cells through the interactions with lipid raft machinery, suggesting a common entry mechanism for the MVs derived from Gram-negative and Gram-positive bacteria. Intact MVs derived from *S. aureus* 06ST1048 isolate induced host cell death in a dose-dependent manner, whereas lysis of MVs or destruction of lipid rafts on host cell membrane resulted in neither the delivery of MV components nor induced cytotoxicity. This finding suggests that the entry of MV components into the cytosol of host cells is prerequisite for cytotoxicity. Although we identified proteins packaged in the *S. aureus* MVs, specific cytotoxic molecules were not characterized in this study. Further studies into the identification of specific virulence factors that are responsible for host cell damage packaged in the MVs and immune response against MVs are expected to provide insights into the association of *S. aureus* pathogenesis and disease progression.

In conclusion, our study demonstrates that *S. aureus* MVs, like OMVs from Gram-negative bacteria, provide an important vehicle for the delivery of bacterial effector molecules to host cells. To the best of our knowledge, this work represents the first study to demonstrate the entry mechanism and cytotoxicity of *S. aureus* MVs in host cells. Our findings have significant implications for the study of Gram-positive bacterial pathogenesis and the development of a new therapeutic target against *S. aureus*.

## Materials and Methods

### Bacterial strain and cell culture

Four *S. aureus* strains, two type strains (ATCC 25923 and ATCC 700699) and two clinical isolates (TSST-1 producing *S. aureus* 103D and MRSA 06ST1048), were used in this study. *S. aureus* 06ST1048 that was isolated from the blood of a hospitalized patient at the Kyungpook National University Hospital in Daegu, Korea and *S. aureus* 103D that was isolated from the skin of atopic dermatitis at the same hospital were obtained from National Culture Collection for Pathogens-Kyungpook National University Hospital (Daegu, Korea). *S. aureus* 06ST1048 is resistant to oxacillin (minimum inhibitory concentration of oxacillin, > 512 µg/ml) and *S. aureus* 103D carries TSST-1 gene. *S. aureus* was identified using both the Vitek Auto Microbic System (BioMérieux Vitek Systems Inc.) and API kit (bioMerieux, Marcy l'Etoile, France). The organisms were maintained on blood agar plates and cultured in LB broth. HEp-2 cells derived from human laryngeal carcinoma were obtained from Korean Cell Line Bank (Seoul, Korea) and were grown in Dulbecco's modified Eagle's medium (HyClone) supplemented with 10% fetal bovine serum (HyClone), 2 mM _L_-glutamine, 1,000 U/ml penicillin G, and 50 µg/ml streptomycin at 37°C in 5% CO_2_.

### Purification of MVs from culture supernatants

The extracellular vesicles of *S. aureus* were prepared from bacterial culture supernatants using a method adapted from Wai *et al*. [14]. *S. aureus* 06ST1048 was inoculated into 500 ml of LB broth and grown until the optical density at 600 nm (OD_600_) reached 1.0 at 37°C with shaking. After bacterial cells were removed by centrifugation at 6000 *g* for 15 min, the supernatants were filtered through a 0.2 µm hollow fiber membrane (GE Healthcare) to remove residual bacteria and cellular debris, and they were then concentrated by ultrafiltration with a QuixStand Benchtop System (GE Healthcare) using a 100 kDa hollow fiber membrane (GE Healthcare). The MV fractions were centrifuged at 150,000 *g* for 3 h at 4°C, and the pellets containing MVs were resuspended in phosphate-buffered saline (PBS). The samples were layered over a sucrose gradient (1.25 ml each of 2.5, 1.6, and 0.6 M sucrose) and centrifuged at 200,000 *g* for 20 h at 4°C; four fractions of equal volumes were collected from the bottom. Sucrose was removed by ultracentrifugation at 150,000 *g* for 3 h at 4°C, and the purified MVs were resuspended in PBS. The sucrose density and protein concentration were determined using refractometry and the Bradford assay (BioRad Laboratories), respectively. The protein concentration was determined using the modified BCA assay (Thermo Scientific). The purified MVs were checked for sterility and stored at −80°C until used.

### SDS-PAGE and Western blot analysis

The purified MVs were resuspended in SDS-PAGE sample buffer (1M Tris HCl pH 6.8, 10% SDS, 1% bromophenol blue, glycerol, and β-mercaptoethanol) and boiled for 10 min. A sample corresponding to 10 µg of protein concentration was separated by 12% SDS-PAGE, and the gels were stained with Coomassie Brilliant Blue R-250 (Bio-Rad). The proteins on the gels were transferred to a nitrocellulose membrane (Amersham Pharmacia Biotech) and then analyzed by Western blotting using rabbit anti-protein A antibodies (Sigma life Science) and mouse anti-β-lactamase immune sera, which were produced in our laboratory. The membrane was incubated with a secondary antibody coupled to horseradish peroxidase and developed by an enhanced chemiluminescence system (ECL plus; Amersham Pharmacia Biotech).

### Identification of proteins in *S. aureus* MVs

One-dimensional electrophoresis-liquid chromatography-tandem mass spectrometry (1-DE-LC-MS/MS) was performed to identify proteins packaged in the *S. aureus* MVs. Proteins were separated via 12% SDS-PAGE and in-gel digested. The protein digests were resolved in 15 µl of 0.02% formic acid in 0.5% acetic acid, and the samples were concentrated on a MGU30-C18 trapping column (LC Packings) and a nano-column (C18 reverse-phase column, Proxeon). The peptides were eluted by 0–65% acetonitrile for 80 min. All MS and MS/MS spectra in the LCQ-Deca ESI ion trap mass spectrometer were acquired in data-dependent mode. The MS/MS spectra were searched using MASCOT software (Matrix Science, Inc.) using the genome data of *S. aureus* TW20 from NCBInr (http://www.ncbi.nlm.nih.gov/) and the decoy sequence database. The exponentially modified protein abundance index (emPAI) was generated using MASCOT software [39].

### TEM analysis

The purified MV samples were applied to copper grids (Electron microscopy sciences, Hatfield, PA) and stained with 2% uranyl acetate. The lung tissues were fixed with 2.5% glutaraldehyde and post-fixed in 1% osmium tetroxide. The samples were dehydrated in a series of ethanol concentrations and embedded in Epon. Thin sections were cut with an ultramicrotome (RMC Boeckeler Instruments) equipped with a diamond knife and stained with 3% uranyl acetate and lead citrate. The samples were then visualized with a TEM (Hitachi H-7500, Hitachi, Japan) operated at 120 kV.

### Confocal microscopy

The cultured HEp-2 cells were seeded at a density of 5×10^4^ on glass coverslips. After treating the MVs, cells were washed with PBS, fixed in 4% paraformaldehyde, and permeabilized for 10 min in PBS containing 0.25% Triton X-100. The MVs were labeled with an anti-rabbit protein A antibody, followed by Alexa-488-conjugated goat anti-rabbit antibody (Molecular Probes). The nuclei of the cells were stained with 4′, 6-diamidino-2-phenyllindole dihydrochloride (DAPI) (Molecular Probes). HEp-2 cells were treated with 10 mM MβCD (Sigma-Aldrich) to disrupt cholesterol-rich membrane domains for 45 min at 37°C in a CO_2_ incubator. After washing with PBS, cells were treated with MVs. The samples were observed with a Carl-Zeiss confocal fluorescent microscope.

### Flow cytometric analysis

The cells were treated with various concentrations of MVs for 24 h and then stained with fluorescein isothiocyanate (FITC)-conjugated Annexin V and PI (BD Pharmingen) according to the manufacturer's instructions. The cells were stained with FITC-conjugated Annexin V in Annexin V binding buffer for 15 min. PI was added to determine alterations in cell membrane integrity. To determine the cytotoxic effect of lysed MVs, the purified MVs were lysed with 0.1M EDTA at 37°C for 1 h and then treated to HEp-2 cells for 24 h. The samples were immediately analyzed by the flow cytometry and CellQuest Pro software (BD Biosciences). For each sample, 10^4^ cells were acquired for data analysis.

### Mouse pneumonia model

Eight-week-old female C57BL/6 mice were used in this study. Immunocompromised mice were infected with *S. aureus* 06ST1048. Neutropenic mice were induced via intraperitoneal injections of cyclophosphamide (150 mg/kg) on days -4 and -3 before bacterial injection [22], [40]. The mice with neutropenia were anesthetized with pentobarbital and then 100 µl of 1×10^8^ cfu/ml of bacteria were administered intratracheally. The control mice were injected with 100 µl of PBS (pH 7.4). The mice were sacrificed 18 h after bacterial challenge, and the lungs were removed. All procedures involving animals were approved by the Animal Care Committee of Kyungpook National University (KNU2010-40).

### Statistical analysis

The statistical significance of the data was determined by Student's *t*-test. A *p* value of <0.05 was considered to be statistically significant.

## Supporting Information

Table S1 Proteins identified in the membrane vesicles derived from S. aureus 06ST1048 using 1-DE and LC-MS/MS analysis
(XLS)
